# Recent Advances in π-Stacking Interaction-Controlled Asymmetric Synthesis

**DOI:** 10.3390/molecules29071454

**Published:** 2024-03-24

**Authors:** Jiaxi Xu

**Affiliations:** 1 State Key Laboratory of Chemical Resource Engineering, Department of Organic Chemistry, College of Chemistry, Beijing University of Chemical Technology, Beijing 100029, China; jxxu@mail.buct.edu.cn; Tel./Fax: +86-10-6443-5565; 2 College of Science, Henan Agricultural University, Zhengzhou 450002, China

**Keywords:** asymmetric synthesis, asymmetric induction, diastereoselectivity, π-stacking interaction

## Abstract

The π-stacking interaction is one of the most important intramolecular and intermolecular noncovalent interactions in organic chemistry. It plays an important role in stabilizing some structures and transition states in certain reactions via both intramolecular and intermolecular interactions, facilitating different selectivities, such as chemo-, regio-, and stereoselectivities. This minireview focuses on the recent examples of the π-stacking interaction-controlled asymmetric synthesis, including auxiliary-induced asymmetric synthesis, kinetic resolution, asymmetric synthesis of helicenes and heterohelicenes, and multilayer 3D chiral molecules.

## 1. Introduction

Attractive π-stacking interactions between π-systems (both aromatic ring and other conjugated systems, even double and triple bonds) play various important roles in diverse phenomena, including the stabilization of biological macromolecules, such as the helical structures of DNA and tertiary structures of proteins, even the complexation of biomolecules and small organic compounds [1,2,3]; the stabilization of the complexation in host–guest systems [4,5]; and controlling selectivities in organic reactions [6,7,8,9]. They can not only control chemoselectivity [10,11,12,13,14] and regioselectivity [15,16,17,18,19] but also stereoselectivities, including diastereoselectivity and enantioselectivity, in diverse organic reactions [20,21]. In 1995, Jones and Chapman wrote a comprehensive review on the π-stacking effect in asymmetric synthesis [20]. π-stacking effects in chiral auxiliary-controlled asymmetric synthesis have been summarized. The auxiliaries include cyclohexane-based arenecarbonyl, arylacetyl, *N*-arylcarboxamide, and aryl carboxylate auxiliaries; 4-aryl/arylmethyloxazolidinone-based auxiliaries; axial chirality auxiliaries; natural product-based auxiliaries; and so on [20]. In 2007, Yamada summarized the intramolecular cation–π interaction in organic synthesis in his perspective [22]. In 2010, Xu collected the most important examples of the influence of the electronic effect of catalysts on the enantioselectivity in asymmetric catalytic organic reactions [21]. Attractive noncovalent interactions, especially hydrogen bonding between the ligand and substrate in enantioselective transition metal catalysis, were reviewed in 2020 [23]. Since 1995, some new chiral auxiliaries have been developed and applied in the π-stacking interaction-controlled asymmetric synthesis. Particularly, during the last two decades, the π-stacking interaction has also been applied in the preparation of optically active (hetero)helicenes and multilayer three-dimensional (3D) chiral molecules as potential materials. This minireview focuses on new developments in the π-stacking interaction-controlled asymmetric synthesis, including several newly developed auxiliary-induced asymmetric syntheses, kinetic resolution for the asymmetric syntheses, and asymmetric syntheses of (hetero)helicenes and multilayer 3D chiral molecules as potential organic materials from 1995 to now. All collected examples in this minireview are mentioned or verified by experimental or theoretical calculational results or on the basis of X-ray crystal diffraction analysis.

## 2. Chiral Auxiliary-Induced Asymmetric Synthesis

### 2.1. Chiral Arylsulfinyl-Based Auxiliaries in Asymmetric Synthesis

Enantiomerically pure aryl methyl sulfoxides and diaryl sulfoxides are a class of readily available optically active compounds and have been applied as chiral auxiliary starting materials in various asymmetric syntheses, such as syntheses of optically active fluorinated structurally diverse amines, fluorinated α-amino acids and β-amino alcohols [24], and fluorinated and non-fluorinated heterocyclic compounds [25]. The *ortho*-substituted 2-alkylphenyl 4-methylphenyl sulfoxides were first used as precursors of *ortho*-arylsulfinylbenzylcarbanions in the nucleophilic addition with *N*-(4-methylphenylsulfinyl) or *N*-(4-methylphenylsulfonyl) (tosyl) aldimines and ketimines, thereby affording 4-methylphenylsulfinamides and 4-methylphenylsulfonamides as direct addition products [26,27]. The removal of the *N*-tosyl group was actually not a readily available process and was even inefficient in some cases. To make the synthetic strategy more practical and useful in synthetic organic chemistry, both enantiomerically pure aryl methyl sulfoxides and diaryl sulfoxides as readily available optically active compounds have been further applied in various nucleophilic additions of *N*-(4-methoxyphenyl) aldimines and ketimines, giving rise to *N*-(4-methoxyphenyl)-derived amine derivatives, which are readily and efficiently removed via oxidation. Thus, the nucleophilic addition of both enantiomerically pure aryl methyl sulfoxides and diaryl sulfoxides with *N*-(4-methoxyphenyl) aldimines and ketimines has been well investigated and applied in diverse asymmetric syntheses, such as the synthesis of optically active fluorinated structurally diverse amines, fluorinated α-amino acids and β-amino alcohols [24], and fluorinated and non-fluorinated heterocyclic compounds [25].

**Scheme 1:**
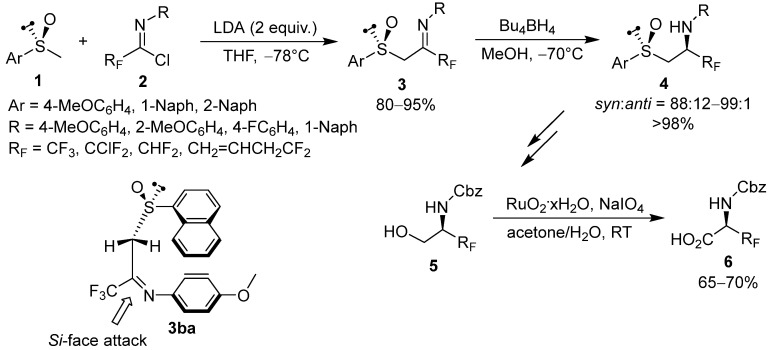
Synthesis of optically active fluorinated α-amino acids and β-amino alcohols.

Peptides have a very wide range of functions in the human body and are a class of widely applied macromolecular medicines. However, they generally survive biodegradation in the human body. To circumvent this biodegradation problem, some structurally similar unnatural peptide analogues are prepared instead of naturally occurring amino acids with non-naturally occurring ones. Fluorinated α-amino acids are one of the choices because fluoro-organic compounds have unique properties, such as lipophilicity, membrane permeability, metabolic stability, and bioavailability. Enantiopure aryl methyl sulfoxides were used in the synthesis of both fluorinated α-amino acids and β-amino alcohols. Enantiomerically pure aryl methyl sulfoxides **1** were first treated with LDA and reacted with fluorinated imidoyl chlorides **2** to form fluorinated chiral arylsulfinyl-derived imines **3**, which were further reduced with tetrabutylammonium borohydride (Bu_4_BH_4_) in MeOH, affording chiral arylsulfinyl-derived amines **4** in almost quantitative yields with excellent diastereoselectivity. In the reduction step, Bu_4_BH_4_ nucleophilically attacked the imines **3** from their *Si*-face due to the existence of the π-stacking interaction between the *N*-aryl group of imines and the *S*-aryl group of sulfoxides. The *Re*-face was shielded by the *S*-aryl group of sulfoxides. After the non-oxidative Pummerer reaction, the obtained optically active fluorinated arylsulfinyl-derived amines **4** were further converted into the corresponding fluorinated β-amino alcohols **5**, which were finally transformed into the desired fluorinated α-amino acids **6** in 65–70% yields via the Ru-catalyzed oxidation with NaIO_4_ as the oxidant (Scheme 1) [24]. The current route is a convenient and useful method for the synthesis of 3,3-difluoro-, 3,3,3-trifluoro-, and 3-chloro-3,3-difluoro-derived alanines.

**Scheme 2:**
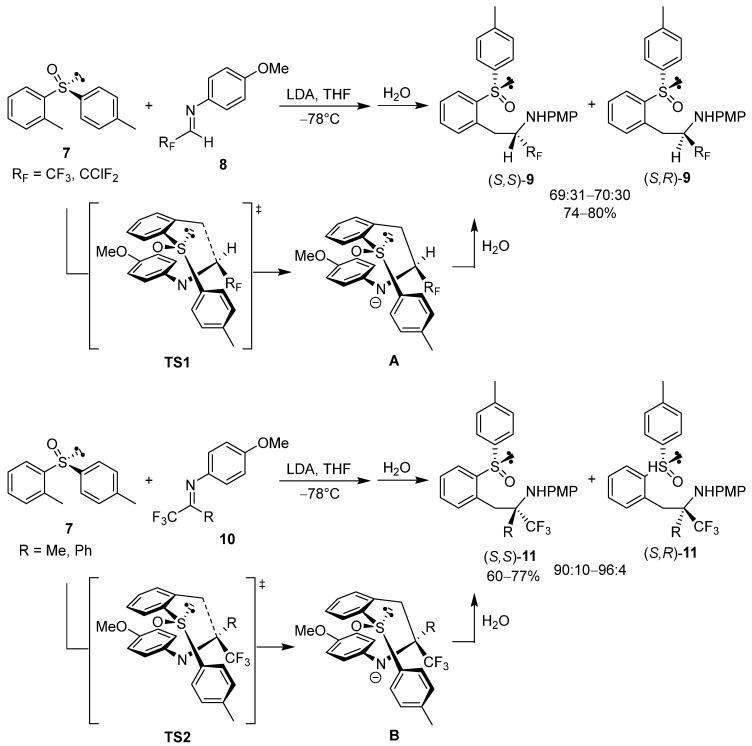
Synthesis of optically active fluorinated sulfinyl-derived amines from aldimines (**A**) and ketimines (**B**).

Enantiomerically pure (*S*)-4-methylphenyl 2-methylphenyl sulfoxide (**7**) was developed as a chiral auxiliary. It was first treated with LDA and reacted with fluorinated aldimines **8** with the *N*-(4-methoxyphenyl) group to form fluorinated chiral arylsulfinyl derived amines (*S*,*S*)-**9** and (*S*,*R*)-**9** in good yields (74–80%) with moderate stereoselectivities (69:31 to 70:30) after workup. The π–stacking interaction between the *N*-(4-methoxyphenyl) group of aldimines **8** and the *S*-aryl group of sulfoxide **7** in both transition states **TS1** and intermediates **A** played a crucial role in controlling the stereoselectivity. However, when (*S*)-4-methylphenyl 2-methylphenyl sulfoxide (**7**) was treated with LDA and then reacted with fluorinated ketimines **10** with the *N*-(4-methoxyphenyl) group to generate the corresponding fluorinated chiral arylsulfinyl derived amines (*S*,*S*)-**11** as major products in satisfactory yields (60–77%) with excellent stereoselectivities ((*S*,*S*)-**11**:(*S*,*R*)-**11** = 90:10 to 96:4). The π–stacking interaction between the *N*-(4-methoxyphenyl) group of ketimines **10** and the *S*-aryl group of sulfoxide **7** in both transition states **TS2** and intermediates **B** also played a key role in controlling the stereoselectivity. The results indicated that ketimines showed better stereoselectivities than aldimines (Scheme 2) [24]. The *ortho*-substituted benzylcarbanion with chiral arylsulfinyl auxiliary improved the stereoselectivity efficiently.

**Scheme 3:**
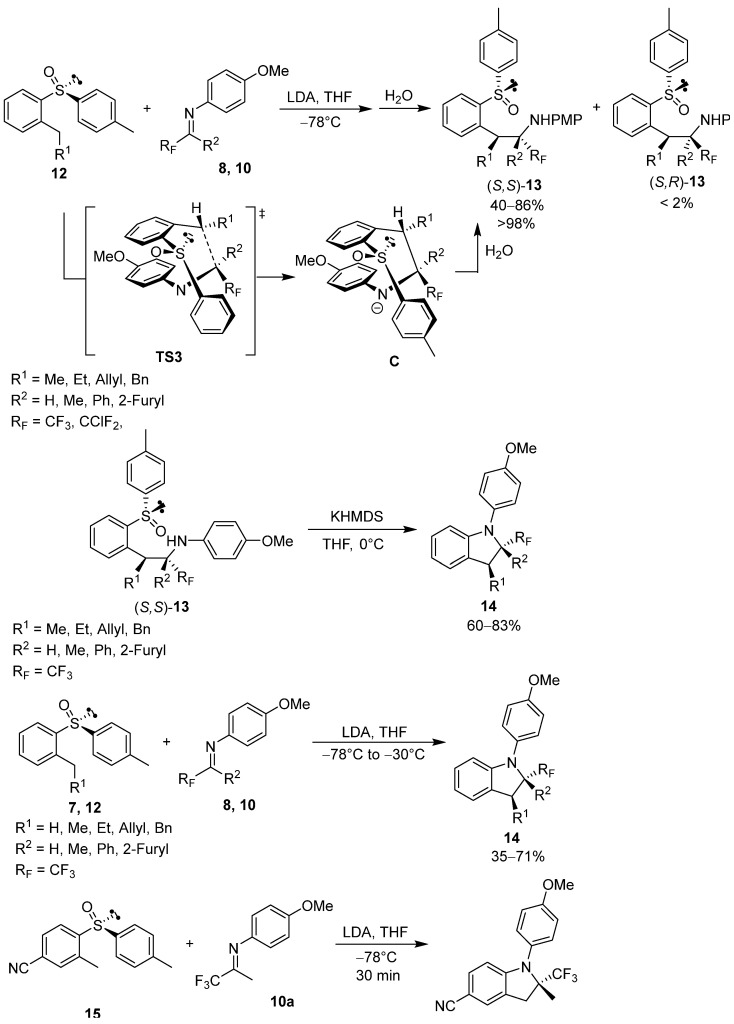
Synthesis of optically active fluorinated sulfinyl-derived amines and indolines.

To further extend the application of *ortho*-substituted benzylcarbanions with chiral arylsulfinyl auxiliary in the stereoselective nucleophilic addition of various imines, enantiomerically pure (*S*)-4-methylphenyl 2-alkylaryl sulfoxides **12** were also developed as chiral auxiliaries. After the treatment with LDA, they reacted with both fluorinated aldimines **8** and ketimines **10** to give rise to the corresponding fluorinated chiral arylsulfinyl-derived amines (*S*,*S*)-**13** in moderate to good yields (40–86%) with excellent stereoselectivities (>98%) after workup. The similar π-stacking interaction in both transition states **TS3** and intermediates **C** controlled the stereoselectivity almost completely. If fluorinated arylsulfinyl-derived amines (*S*,*S*)-**13** were further treated with KHMDS in THF at 0 °C, they underwent an intramolecular aromatic nucleophilic substitution with arylsulfinyl groups as the leaving groups, affording the corresponding fluorinated indoline derivatives **14** in 60–83% yields. Only trifluoromethyl-derived products (*S*,*S*)-**13** (R_F_ = CF_3_) were tested. The results illustrated that indoline derivatives **14** could be generated in one pot. The tandem reaction was attempted. After the nucleophilic addition of 2-(4-methylphenylsulfinyl)benzylcarbanions and aldimines **8** or ketimines **10** at −78 °C, the reaction mixture was warmed to −30 °C and continually stirred for 30 min. Further intramolecular aromatic nucleophilic substitution occurred, producing the desired fluorinated indoline derivatives **14** in 35–71% yields via tandem nucleophilic addition and intramolecular aromatic nucleophilic substitution in one pot, exhibiting high step-economy. In comparison with the step-wise synthetic method, the yields in the tandem fashion were similar to the total yields for two steps in the step-wise route. Furthermore, the one-pot tandem reaction of (*S*)-4-methylphenyl 4-cyano-2-methylphenyl sulfoxide (**16**) with a cyano functional substituent and trifluoromethyl ketimine **10a** was performed with LDA as a base in THF at −78 °C; the desired indoline derivative **16** was obtained in 45% yield after stirring for 30 min. The cyano group survived in the tandem reaction in the presence of *ortho*-arylsulfinylbenzylcarbanion as the strong nucleophile, showing good functional group tolerance (Scheme 3) [25].

**Scheme 4:**
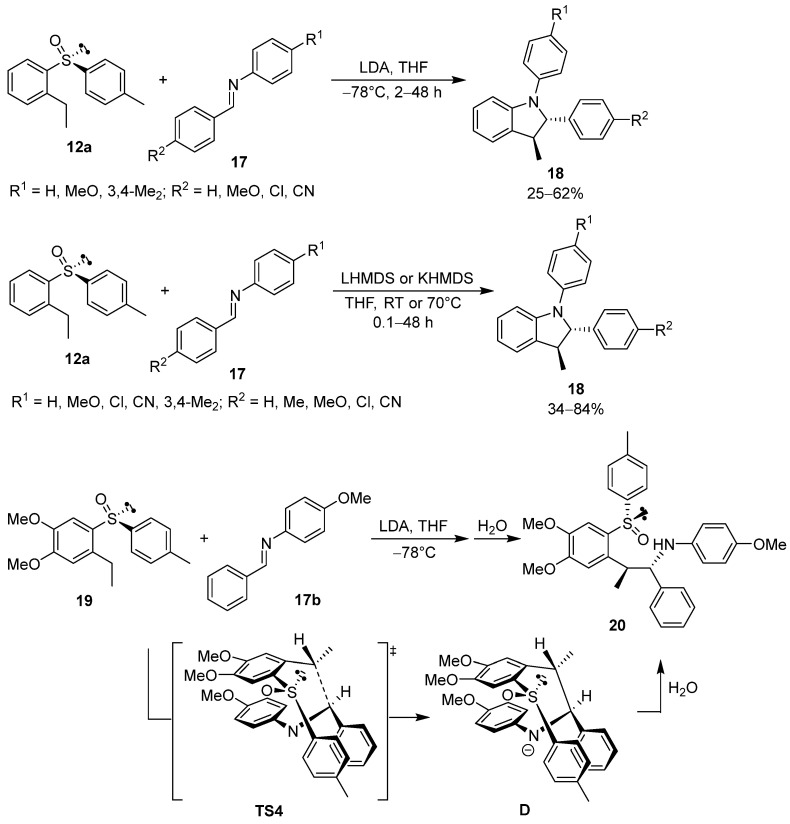
Synthesis of optically active indolines.

The indoline skeleton is a ubiquitous moiety in the structures of many alkaloids and natural products. Indolines are generally considered to be key privileged structures for their diverse biological activities. To develop a step-economic and efficient asymmetric synthetic method of biologically important and optically active indoline derivatives, the above developed strategy was extended to the synthesis of non-fluorinated indoline derivatives via tandem nucleophilic addition and intramolecular aromatic nucleophilic substitution with the 4-methylphenylsulfinyl group as the leaving group. When fluorinated aldimines **8** and ketimines **10** were displaced with aromatic imines **17** generated from aromatic aldehydes and aromatic amines, the reaction of (*S*)-(2-ethylphenyl) 4-methylphenyl sulfoxide (**12a**) and aromatic imines **17** generated (2*S*,3*S*)-2,3-diaryl-4-methylindolines **18** in moderate to satisfactory yields of 25–62% with LDA as a base. When LHDMS or KHMDS was used as the base instead of LDA, the reaction was conducted at room temperature or at 70 °C to give the corresponding products **18** in higher yields than those with LDA as the base. The aromatic imines with electron-withdrawing substituents generally required longer reaction times and higher reaction temperatures than those with electron-donating groups. The reaction of electron-rich (*S*)-1-ethyl-4,5-dimethoxy-2-(4-tolylsulfinyl)benzene (**19**) and (*E*)-*N*-(4-methoxyphenyl)-1-phenylmethanimine (**17b**) stopped at the nucleophilic addition step in the presence of LDA as the base, generating the corresponding amine **20** as the final product after workup, rather than the desired indoline derivative because the electron-rich arylsulfinyl with two strong electron-donating methoxy groups could not undergo the intramolecular aromatic nucleophilic substitution. Upon further treatment of the amine **20** with LHMDS, no reaction occurred as well, indicating that the electron-rich substrate indeed hardly underwent the intramolecular aromatic nucleophilic substitution even in a step-wise fashion. For each of these cases, the π-stacking interaction between the *N*-aryl group of imines and the *S*-aryl group of sulfoxides in both the transition state **TS4** and intermediate state **D** played an important role in controlling the stereoselectivity (Scheme 4) [28].

### 2.2. Adducts of Levoglucosenone and 9-(Aryloxymethyl)arthracenes as Chiral Auxiliaries in Asymmetric Synthesis

**Scheme 5:**
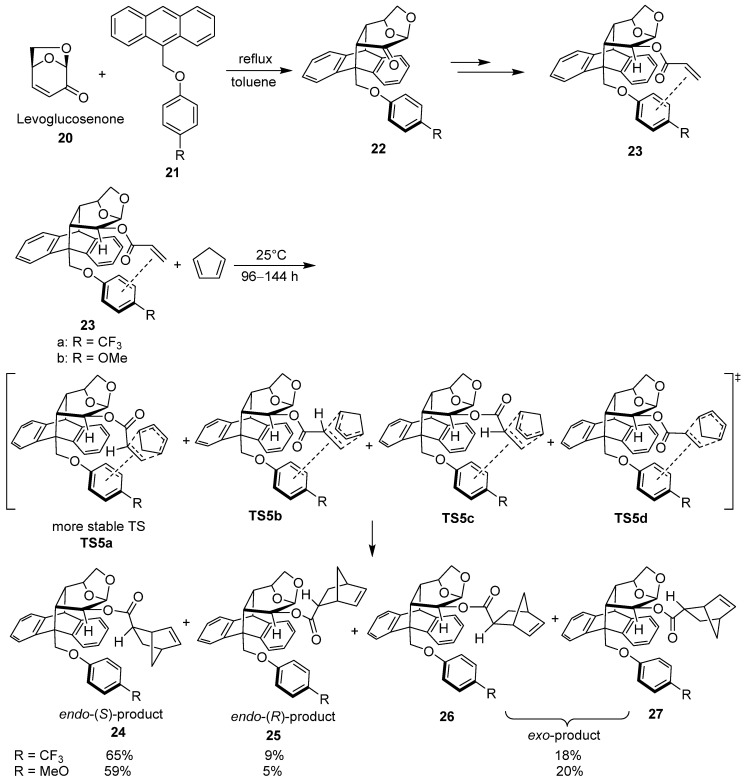
π-stacking-controlled Diels–Alder reaction with *endo*-(*S*)-products as major products.

The Diels–Alder cycloaddition of alkyl acrylates and cyclopentadiene can generate both *endo*-adducts and *exo*-adducts. If chiral alkyl acrylates were utilized, asymmetric induction would occur. Acrylate derivatives **23** bearing *para*-trifluoromethyl and methoxyphenoxymethyl substituents as the π-stacking templets and shelter were prepared via the Diels–Alder reaction of enantiomerically pure levoglucosenone (**20**) and 9-(*para*-trifluoromethyl and methoxyphenoxymethyl)arthracenes (**21**), and the subsequent reduction and acrylation. There is an intramolecular vinyl-aryl π-stacking interaction between the acrylate and aryloxy groups. When they were applied in the Diels–Alder reaction with cyclopentadiene, evident π-stacking-controlled asymmetric synthesis was observed, generating *endo*-(*S*)-bicyclo [2.2.1]hept-5-ene-2-carboxylates **24** in 65% and 59% yields as major products through more the stable transition state **TS5a**. Cyclopentadiene would approach the C=C bond in the acrylate moiety only from its top direction in all transition states because the aryloxy group was fixed below the C=C bond due to the existence of the vinyl-aryl π-stacking interaction between the acrylate and aryloxy groups (Scheme 5) [29]. 

**Scheme 6:**
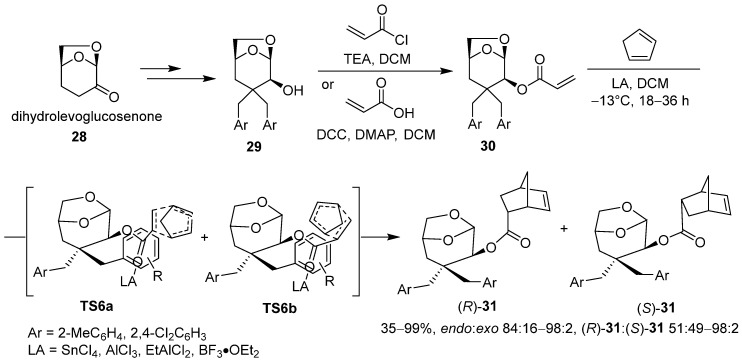
π-stacking-controlled Diels–Alder reaction with *endo*-(*R*)-products as major products.

Both enantiomerically pure levoglucosenone (**20**) and its dihydro derivative **28** are readily available from biomass because they are products of cellulose pyrolysis. Enantiomerically pure dihydrolevoglucosenone (**28**) was also applied as a chiral auxiliary in the diastereoselective Diels–Alder reaction. It was first converted to dibenzylated dihydrolevoglucosenols (**29**) through double benzylation with benzyl halides under basic conditions followed by a reduction with sodium borohydride. Differently, dibenzylated dihydrolevoglucosenols (**29**) were further acrylated and then reacted with cyclopentadiene in the presence of Lewis acids in DCM, affording *endo*-(*R*)-bicyclo [2.2.1]hept-5-ene-2-carboxylates (*R*)-**31** as major products due to the existence of the vinyl-aryl π-stacking interaction (Scheme 6) [30]. Through the utilization of both levoglucosenone (**20**) and its dihydro derivative **28** as auxiliaries, both enantiomeric bicyclo [2.2.1]hept-5-ene-2-carboxylates were prepared in good to high yields. Both diastereomeric monobenzylated dihydro derivatives were also attempted as auxiliaries in the diastereoselective Diels–Alder reactions. They show excellent *endo*/*exo* selectivity, but their *R*/*S* stereoselectivity is generally lower than the corresponding dibenzylated dihydrolevoglucosenone (**28**). 

### 2.3. Chiral Oxazolidinone-Based Auxiliaries in Asymmetric Synthesis

**Scheme 7:**
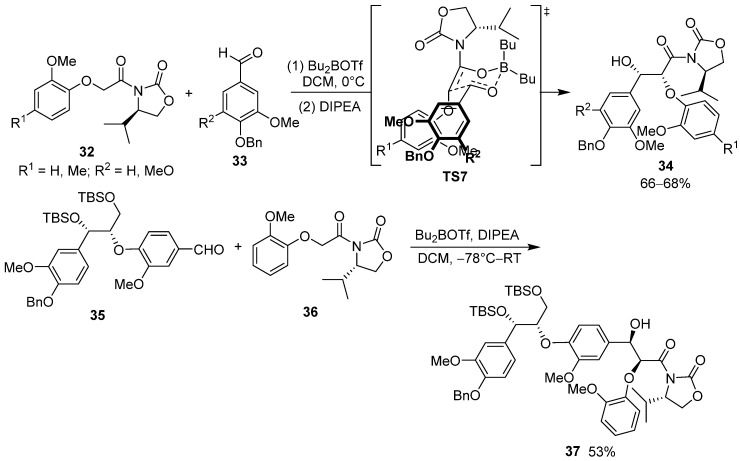
Asymmetric synthesis of dimers and trimer of lignin models.

Lignin is a class of natural plant-based polymers and ranks second in abundance only after cellulose, making it a potentially valuable raw material for biorefinery. However, it is a considerable challenge to use lignin as a feedstock for the production of biobased chemicals in either catalytic or enzymatic processes due to the structural heterogeneity of lignin. The heterogeneity is the result of the biosynthesis of lignin from the radical coupling of three primary monolignols. To improve lignin’s utility as a renewable carbon feedstock, it is necessary to understand the assembly, stereostructure, and reactivity of the separation of lignin and the enzymatic lignin disassembly process. To realize these purposes, it is required to synthesize lignin models with different configurations in a stereospecific manner. To enantioselectively synthesize lignin models, Njiojob and coworkers selected the Evans auxiliary as the chiral source. They first prepared *N*-(2-methoxyphenyloxy)acetylated (*R*)-4-isopropyloxazolidin-2-ones **32** as starting materials. The reaction of aryloxyacetyl-derived (*R*)-4-isopropyloxazolidin-2-ones **32** and 4-benzyloxybenzaldehydes **33** stereospecifically generated lignin dimer models **34** in 60–68% yields in the presence of di-*n*-butylboron triflate and diisopropylethylamine (DIPEA) via condensation through six-membered Zimmerman-Traxler transition states **TS7**, in which the π-stacking interaction between benzaldehydes and aryloxy groups plays an important role in controlling the stereoselectivity. After subsequent transformations, including reduction, protection of the hydroxyl group, and oxidation, one of the lignin dimer models **34** was converted into aldehyde **35**. Following a similar strategy, the reaction of aldehyde **35** with (*S*)-4-isopropyl-*N*-(2-methoxyphenyloxy)acetyloxazolidin-2-ones **36** as the chiral starting material, a lignin trimer model **38** was synthesized in a 53% yield. (*R*)- and (*S*)-Evans auxiliaries **32** and **36** show completely opposite stereoselectivities (Scheme 7) [31]. The current synthetic strategy is an efficient way to prepare enantiopure lignin dimers and trimers with different stereochemical configurations from aryloxyacetylate oxazolidin-2-ones derivatives and appropriate aromatic aldehydes.

## 3. Acyl 2,3-Dihydroimidazo[1,2-*a*]pyridine and 1,2-Dihydroimidazo[1,2-*a*]quinolines in Kinetic Resolution

Although the asymmetric catalytic borane reduction of ketones is one of the most efficient methods for the preparation of enantiopure secondary alcohols [32,33,34], chemical resolution has been widely utilized in the industrial manufacturing of secondary alcohols as well [35,36]. Kinetic resolution is also an alternative choice for obtaining enantiopure secondary alcohols via selected reactions, for instance, acylation [37,38].

**Scheme 8:**
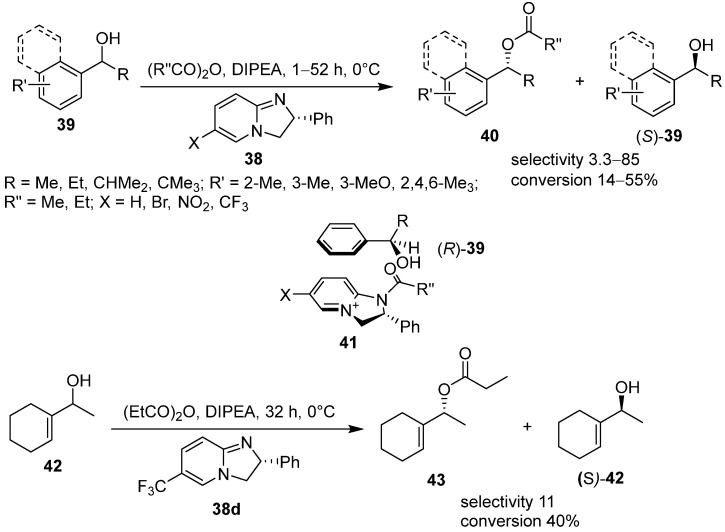
Synthesis of phosphonopeptides with DCC as a coupling reagent.

Birman’s group first developed 6-substituted (*R*)-2-phenyl-2,3-dihydroimidazo[1,2-*a*]pyridines **38** as enantioselective acylation catalysts. In the resolution, the catalysts first were acylated with acetic or propanoic anhydrides in the presence of DIPEA. The acylated 2,3-dihydro-1*H*-imidazo[1,2-*a*]pyridin-4-iums **41** predominantly reacted with (*R*)-alcohols (*R*)-**39** due to less steric hindrance in the π-stacking attractive interaction. Generally, non-substituted and 6-bromo-2,3-dihydroimidazo[1,2-*a*]pyridines (**38a** and **38b**) exhibited lower selectivities than 6-nitro- and 6-trifloromethyl-2,3-dihydroimidazo[1,2-*a*]pyridines (**38c** and **38d**). Bulky alcohols (R = CHMe_2_ and CMe_3_) generally required long reaction times (30–52 h) to reach a similar conversion. However, aliphatic 1-cyclohexylethan-1-ol showed only less than 4% conversion for 50 h due to the absence of the π-stacking interaction. Although indan-1-ol was converted in 16% for 50 h, no selectivity was observed because of the nonexistence of the π-stacking interaction. These results support the function of the π-stacking attractive interaction in the kinetic resolution. The C=C double bond containing substrate 1-(cyclohexen-1-yl)ethan-1-ol (**42**) was resoluted in a moderate selectivity of 11 in 40% conversion (Scheme 8) [37].

**Scheme 9:**
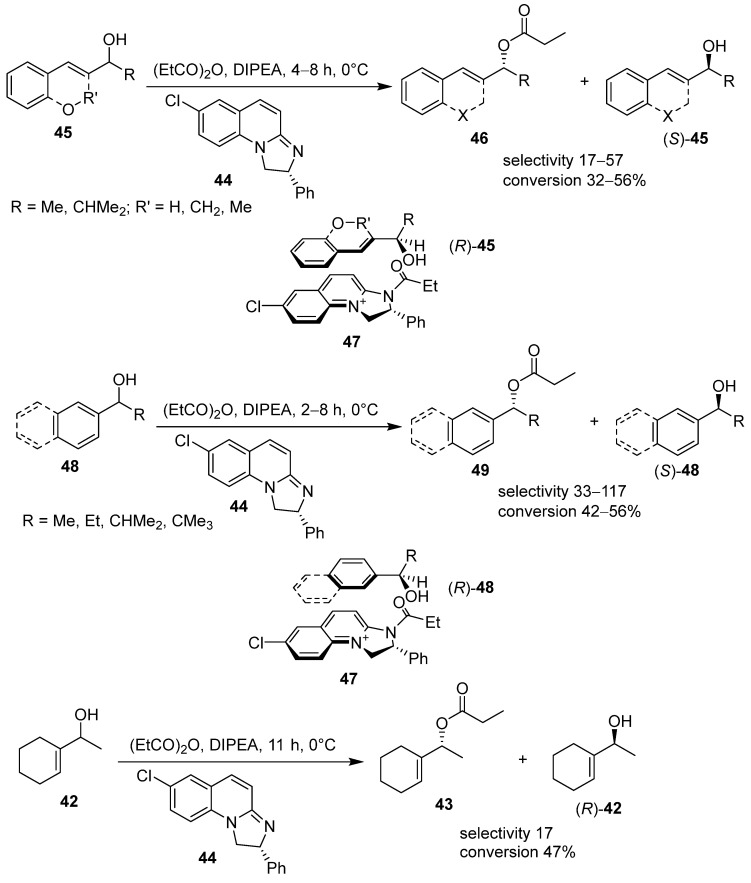
Kinetic resolution of alcohols with 1,2-dihydroimidazo[1,2-*a*]quinolone as the catalyst.

To realize the kinetic resolution of conjugated cinnamyl alcohols and their derivatives **45**, they further developed new (*R*)-7-chloro-2-phenyl-1,2-dihydroimidazo[1,2-*a*]quinoline (**44**) with an additional fused benzene ring as the enantioselective acylation catalyst. In comparison with 2,3-dihydroimidazo[1,2-*a*]pyridines **38**, 1,2-dihydroimidazo[1,2-*a*]quinoline **44** extended the conjugative system and achieved efficient a kinetic resolution of both conjugated cinnamyl alcohols **45** with selectivities of 17–57 in conversions of 32–56% and non-conjugated 1-arylethan-1-ols **48** with selectivities of 33–117 in conversions of 42–56%; even the C=C double bond containing substrate 1-(cyclohexen-1-yl)ethan-1-ol (**42**) with a selectivity of 17 in a conversion of 47% with propanoyl anhydride was applied as the acylating reagent. In each of the cases, (*R*)-alcohols were acylated to the corresponding propanoates and (*S*)-alcohols remained (Scheme 9) [38]. All of these three classes of substrates were efficiently resoluted with (*R*)-7-chloro-2-phenyl-1,2-dihydroimidazo[1,2-*a*]quinoline (**44**) as the catalyst and propanoyl anhydride as the acylating reagent. However, bulky alcohols generally presented high selectivities. For example, 2,2-dimethyl-1-phenylpropan-1-ol was resoluted with the selectivity of 117. The results revealed that 1,2-dihydroimidazo[1,2-*a*]quinoline **44** with an extended conjugative system showed better behavior than 2,3-dihydroimidazo[1,2-*a*]pyridines **38** in the kinetic resolution of alcohols.

## 4. Asymmetric Synthesis of β-Lactams and Deoxygenation of Oxiranecarbonitriles via Intramolecular π–π Stacking Interaction

**Scheme 10:**
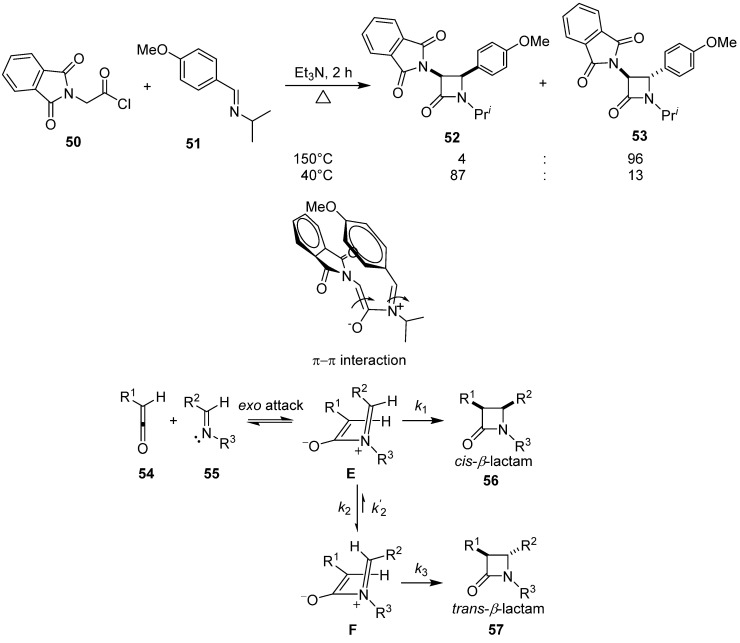
Diastereoselective synthesis of β-lactams.

β-lactam has been a key structural moiety of widely applied β-lactam antibiotics all over the world since the 1940s. β-lactam antibiotics have helped millions of people. Most β-lactam antibiotics are prepared from semisynthesis. The key structures of the β-lactam ring with defined stereostructures are generally constructed by organisms. The stereoselective synthesis of β-lactam derivatives is a crucial issue in constructing the β-lactam ring in both organic and medicinal chemistry [39]. The Staudinger cycloaddition is a versatile method of synthesizing β-lactams from imines and ketenes, generated from acyl chlorides or α-diazoketones [40,41]. The diastereoselectivity in the Staudinger cycloaddition is controlled by the competition between the direct ring closure and isomerization of zwitterionic intermediates **E** generated from imines **55** and ketenes **54** (Scheme 10) [42]. In contrast with other factors [43], the substituents of imines and ketenes and reaction temperature evidently impact diastereoselectivity [44]. On the other hand, torquoselectivity also plays an important role in the diastereocontrol in disubstituted ketene-participating Staudinger cycloaddition [45].

To illustrate the influence of different substituted ketenes and imines on diastereoselectivity in the formation of β-lactams at various reaction temperatures, the reaction of phthalimidoacetyl chloride (**50**) and *N*-(4-methoxybenzylidene)isopropylamine (**51**) was conducted and exhibited an evident increase in temperature on the diastereoselectivity. There was a favorable formation of *trans*-β-lactam product **53** at a higher temperature (150 °C) and a predominant generation of *cis*-β-lactam product **52** at a lower temperature (40 °C). It was rationalized that the strong π-stacking interaction existed between the electron-withdrawing phthalimido and electron-donating 4-methoxyphenyl groups in intermediate **E** at low temperatures and played an important role in stabilizing the intermediate **E**, leading to the formation of *cis*-β-lactam product **52**. However, the stability of the π-stacking interaction decreased along with the increase in the reaction temperature, resulting in the intermediate **E** converting into intermediate **F**, which generated *trans*-β-lactam product **53** (Scheme 10) [44]. The intramolecular π-stacking interaction played an important role in controlling the formation of *cis*-β-lactam product **54** diastereoselectively at low temperatures.

**Scheme 11:**
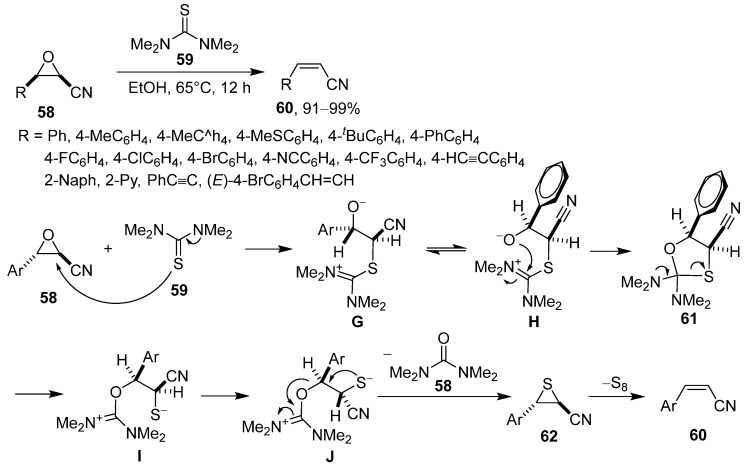
Stereospecific synthesis of (*Z*)-3-arylpropenonitriles from *trans*-3-aryloxiranecarbonitriles.

Oxiranecarbonnitriles are very important synthetic intermediates and have been applied in several transformations [46,47,48,49,50]. During the transformation of 3-substituted *trans*-oxiranecarbonnitriles **58** to 3-substituted (*Z*)-propenonitriles **60** through the thiourea-mediated stereospecific deoxygenation, *trans*-oxiranecarbonnitriles **58** generated (*Z*)-propenonitriles **60** stereospecifically due to the π-stacking interaction in intermediates **G** and **61**. *N*,*N*,*N*′,*N*′-Tetramethylthiourea (**59**) first nucleophilically attacked the cyano-attached ring carbon atom to form intermediates **G** via an S_N_2 ring opening. Intermediates **G** rotated 180° to generate intermediates **H**, which further underwent an intramolecularly nucleophilic addition to produce 1,3-oxathiolane derivatives **61**. In an amine-induced fragmentation, they transformed into intermediates **I**, which rotated 180° to yield intermediates **J**. Intermediates **J** further underwent an intramolecularly nucleophilic substitution, resulting in the formation of *trans*-thiiranecarbonitriles **62** stereospecifically. *Trans*-Thiiranecarbonitriles **62** lost their sulfur atom to lead to (*Z*)-propenonitriles **60** stereospecifically (Scheme 11) [51].

The nucleophilic ring expansion of saturated three-membered heterocycles has been well investigated [52]. Recently, the electrophilic ring expansion of saturated three-membered heterocycles was also realized [18,53,54]. In contrast with the nucleophilic ring expansion of saturated three-membered heterocycles [52], their electrophilic ring expansion is a new avenue to construct new heterocyclic compounds [18,53,54]. The electrophilic ring expansion of polycyclic arylthiiranes **64** and ketenes **K** generated from aryloxyacetyl chlorides **63** in the presence of triethylamine is a new strategy for the synthesis of areno[*d*]-ε-thiolactones **66** directly without catalysts or additives. In the reaction, aryloxyacetyl chlorides **63** are first eliminated in the presence of TEA to form aryloxyketenes **K**.

**Scheme 12:**
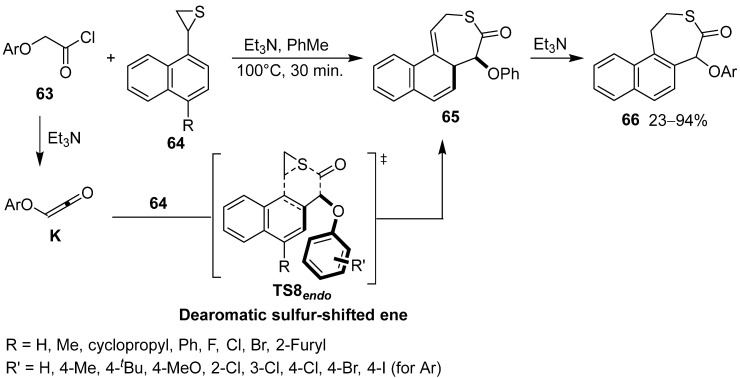
Synthesis of areno[*d*]-ε-thiolactones from arylthiiranes and aryloxyacetyl chlorides.

Arylthiiranes **64** and aryloxyketenes **K** undergo a dearomatic sulfur-shifted ene reaction to directly generate intermediates **66** through the *endo* transition states **TS8*endo*** due to the existence of the π-stacking interaction, which was verified by theoretical calculation. After aromatization, intermediates **65** transform into the final products **66** in 22–94% yields. The current reaction features atom and step-economic characteristics via a tandem sequence of in situ ketene generation, π-stacking-controlled dearomatic sulfur-shifted ene, and aromatization and is a novel strategy for the electrophilic ring expansions of three-membered saturated heterocycles (Scheme 12) [55].

## 5. Asymmetric Synthesis of Optically Active Helicenes

**Scheme 13:**
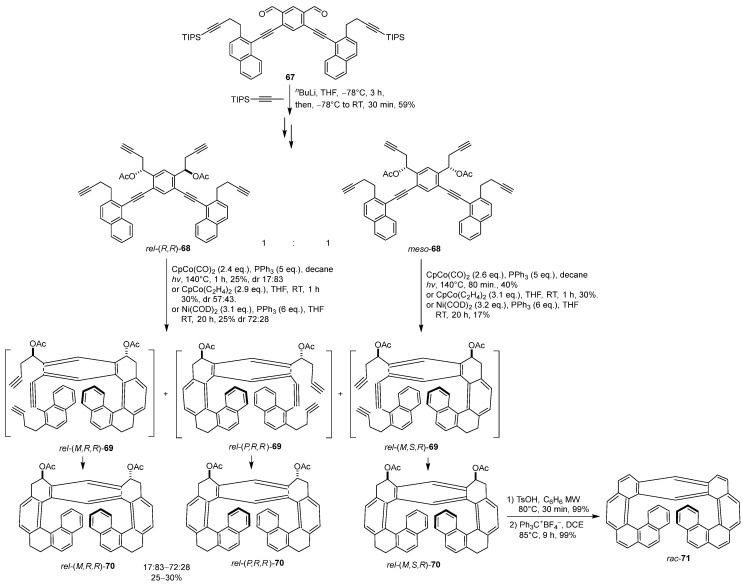
Synthesis of optically active [11]helicene-like molecules.

Helicenes are a class of important carbon-rich materials. Helical supramolecular structures are important in chemistry and materials science. Helical structures that are derived from conjugated *ortho*-annelated arenes or heteroarenes are known as [*n*]helicenes. The n presents the number of the *ortho*-annelated arene or heteroarene ring. Helicenes are 3-dimensional polycyclic aromatic systems, which consist of all *ortho*-fused aromatic or heteroaromatic rings and are inherently chiral, presenting helical conformation. Different from fullerenes, carbon nanotubes, and graphene, helicenes are chiral organic compounds and exist in left-hand or right-hand helical structures. Different racemic [11]helicenes consisting of all *ortho*-fused benzene rings or *ortho*-fused benzene and conjugated cyclohexadiene rings were prepared successfully from 4,6-bis((2-(4-(triisopropylsilyl)but-3-yn-1-yl)naphthalen-1-yl)ethynyl)isophthalaldehyde (**67**), which was synthesized from 4,6-diformyl-1,3-phenylene bis(trifluoromethanesulfonate) and (4-(1-ethynylnaphthalen-2-yl)but-1-yn-1-yl)triisopropylsilane via the CuI-catalyzed coupling. The dialdehyde **67** reacted with *n*-butyl lithium-treated prop-1-yn-1-yltripropylsilane, generating a pair of enantiomeric double addition products and a *meso*-double addition product in an equivalent amount. After a series of transformations, they were converted into diacetates *rel*-(*R*,*R*)-**68** and *meso*-**68** as precursors for the next [2 + 2 + 2] annulation. The [2 + 2 + 2] annulations were conducted under three different conditions. For precursor *rel*-(*R*,*R*)-**68**, it asymmetrically produced double-annulated product [11]helicene-like derivatives *rel*-(*M*,*R*,*R*)-**69** and *rel-*(*P*,*R*,*R*)-**69** through intermediates *rel*-(*M*,*R*,*R*)-**69** and *rel-*(*P*,*R*,*R*)-**69**. The CpCo(CO)_2_-catalyzed annulation preferred the formation of *rel-*(*P*,*R*,*R*)-**70** with a diastereoselectivity of 17:83 for *rel*-(*M*,*R*,*R*)-**70**:*rel-*(*P*,*R*,*R*)-**70**, while the Ni(COD)_2_-catalyzed annulation predominately generated *rel-*(*M*,*R*,*R*)-**70** with a diastereoselectivity of 72:28 for *rel*-(*M*,*R*,*R*)-**70**:*rel-*(*P*,*R*,*R*)-**70**. However, only very low diastereoselectivity was observed in the CpCo(C_2_H_4_)_2_-catalyzed annulation, with a slightly favorable formation of *rel-*(*M*,*R*,*R*)-**70** with a diastereoselectivity of 57:43 for *rel*-(*M*,*R*,*R*)-**70**:*rel-*(*P*,*R*,*R*)-**70**. For the double [2 + 2 + 2] annulation of *meso*-**68**, the Co-catalyzed double [2 + 2 + 2] annulations gave rise to the annulated product [11]helicene-like derivative *rel-*(*M*,*R*,*R*)-**70** in higher yields (40% and 30%) than those in the Ni-catalyzed annulation. The [11]helicene-like derivative *rel-*(*M*,*R*,*R*)-**63** was further converted to racemic [11]helicene *rac*-**70** in excellent yield via the elimination of acetate and dehydrogenation. In all [2 + 2 + 2] annulations, the edge-to-face π-stacking interaction plays a key role in controlling the diastereoselectivity (Scheme 13) [56]. Interestingly, the CpCo(CO)_2_- and Ni(COD)_2_-catalyzed double [2 + 2 + 2] annulations exhibit different diastereoinduction in the stereoselective synthesis of [11]helicene-like molecules.

**Scheme 14:**
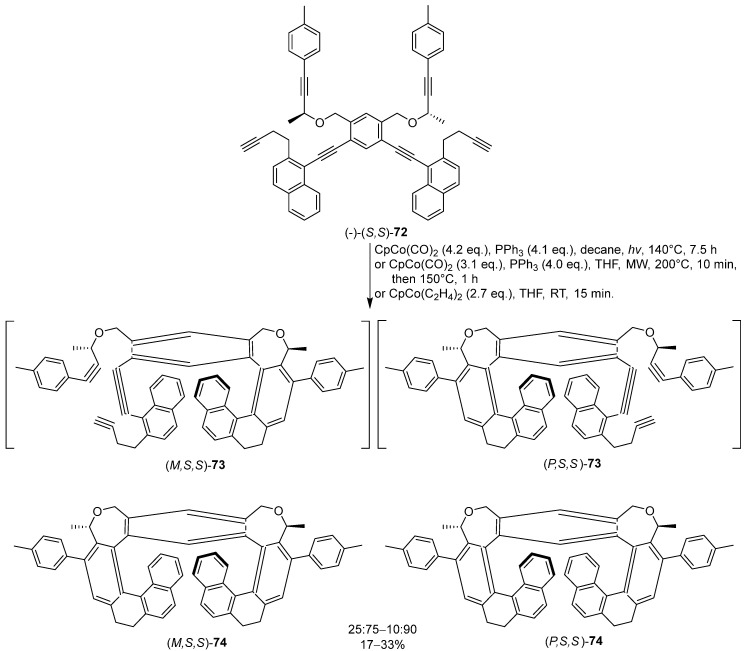
Synthesis of optically active [11]helicene-like molecules.

Helicenes containing one or more nonaromatic heterocycles are also a class of important carbon-rich materials. Similar to helicenes derived from conjugated *ortho*-annelated arenes or heteroarenes, helicene-like molecules also present helical conformation. After the successful preparation of racemic [11]helicenes, the asymmetric synthesis of optically active (*P*,*S*,*S*)- and (*M*,*S*,*S*)-[11]helicene-like molecules (*P*,*S*,*S*)-**74** and (*M*,*S*,*S*)-**74** was realized from 1,1’-((4,6-bis((((S)-4-(*p*-tolyl)but-3-yn-2-yl)oxy)methyl)-1,3-phenylene)bis(ethyne-2,1-diyl))bis(2-(but-3-yn-1-yl)naphthalene) (−)-(*S*,*S*)-**72** through the Co-catalyzed double [2 + 2 + 2] annulation. In the [2 + 2 + 2] annulation, the edge-to-face π-stacking interaction plays a crucial role in controlling the stereoselectivity. Under all the used conditions, (*P*,S,S)-annulated product (*P*,*S*,*S*)-**74** was obtained as the major product. High stereoselectivity ((*M*,*S*,*S*)-**74**:(*P*,*S*,*S*)-**74** = 10:90) was observed under photo-irradiation (in 26% yield) and microwave irradiation (in 33% yield) conditions. However, a low stereoselectivity ((*M*,*S*,*S*)-**74**:(*P*,*S*,*S*)-**74** = 25:75) and yield (17%) were obtained at room temperature. The results indicated that a higher reaction temperature was favorable to improve both the yield and stereoselectivity (Scheme 14) [56]. The current Co-catalyzed double [2 + 2 + 2] annulation strategy is an efficient route to synthesize optically active [11]helicene-like molecules.

**Scheme 15:**
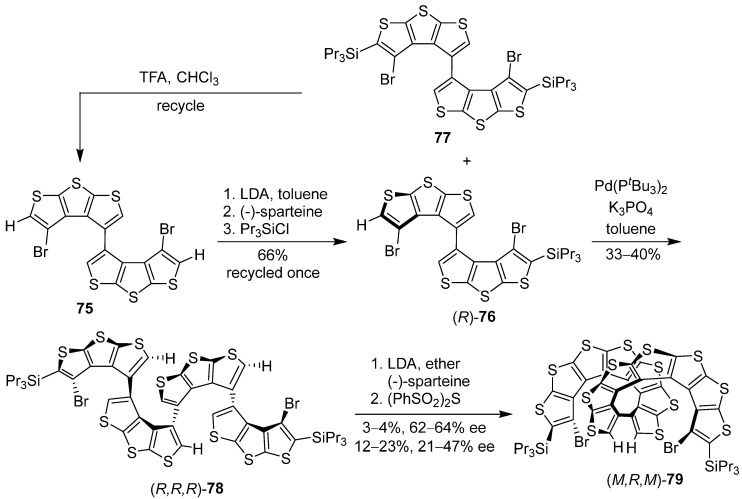
Synthesis of helically locked bis[7]helicene from helically folded tetrakis(β-trithiophene).

Heterohelical supramolecular structures are important in chemistry and materials science as well. Oligothiophenes are the most studied π-systems due to their favorable electrical and optical properties. Much attention has been paid to their preparation. Several classical oligothiophenes, such as α-sexithiophene, quasi-linear oligomer pentathienoacene, and helical oligomer [7]helicene, have been synthesized until now. The asymmetric synthesis of optically active (*M*,*R*,*M*)-bis[7]helicene (*M*,*R*,*M*)-**79** was carried out from bis(β-trithiophene) **75** [57]. Bis(β-trithiophene) **75** was obtained from 3,4-dibromo-2-trimethylsilyldithieno[2,3-*b*:3’,2’-*d*]thiophene via lithiation with butyl lithium and the CuCl_2_-catalyzed coupling followed with the treatment with TFA in dichloromethane. 3,4-Dibromo-2-trimethylsilyldithieno[2,3-*b*:3’,2’-*d*]thiophene was prepared from 3,4-dibromothiophene via lithiation with butyl lithium and the CuCl_2_-catalyzed coupling followed by dilithiation with LDA and oxidation with bis(phenylsulfonyl)sulfide [58]. To circumvent solubility problems, bis(β’-bromo-β-trithiophene) **75** was protected with tripropylsilyl chloride with the treatment with LDA in the presence of a chiral diamine (-)sparteine, affording optically active mono-tripropylsilyl-protected (*R*)-tetrakis(β-trithiophene) (*R*)-**76** and ditripropylsilyl-protected tetrakis(β-trithiophene) **77**, which was hydrolyzed to starting material **75** with TFA in chloroform for reuse. The (*R*)-tetrakis(β-trithiophene) (*R*)-**76** was further coupled under the catalysis of a palladium catalyst in the presence of potassium phosphate to generate tetrakis[7]helicene (*R*,*R*,*R*)-**78** with (*R*,*R*,*R*) configuration. Finally, the (*R*,*R*,*R*)-tetrakis[7]helicene (*R*,*R*,*R*)-78 was transformed to optically active (*M*,*R*,*M*)-bis[7]helicene (*M*,*R*,*M*)-**79** via the formation of two new thiophene rings through the sequential treatments with LDA in the presence of (−)-sparteine and oxidation with bis(benzenesulfonyl) sulfide. During the transformation, the strong noncovalent π–π stacking interaction plays an important role in stereoinduction in the formation of (*M*,*R*,*M*)-bis[7]helicene (*M*,*R*,*M*)-**79**, which showed a helical structure. However, the transformation from (*R*,*R*,*R*)-tetrakis[7]helicene (*R*,*R*,*R*)-**78** into (*M*,*R*,*M*)-bis[7]helicene (*M*,*R*,*M*)-**79** exhibited low to moderate stereoselectivity, 62–64% ee for 3–4% conversion, and 22–47% ee for 12–23% conversion. The stereoselectivity decreased evidently along with the increase in conversion (Scheme 15) [57]. Although the synthetic method is not so efficient in conversion and stereoselectivity, it is still useful for the preparation of optically active bis[7]helicene and will show further application in the preparation of other [n]helicene derivatives in the future.

## 6. Asymmetric Synthesis of Multilayer 3D Chiral Molecules

Multilayer 3D chiral molecules are a new class of macromolecular sandwich-shaped organic materials. They possess a new form of chirality which is different from traditional planar and helical counterparts. They are composed of both planar and axial chirality. Their middle part includes three parallel top, medium, and bottom layers of aromatic (hetero)arene systems, which fold together by an aromatic π-stacking interaction, while the medium aromatic (hetero)arene is linked generally with the top and bottom aromatic arenes, respectively, on its *para*-positions through two naphthalene derivatives, existing in axial chirality. They show a strong luminescence of different colors under UV irradiation and some of them display aggregation-induced emission (AIE) properties. Thus, they exhibit potential applications in chemical, medicinal, and material sciences including optoelectronic materials in the future [59,60]. 

**Scheme 16:**
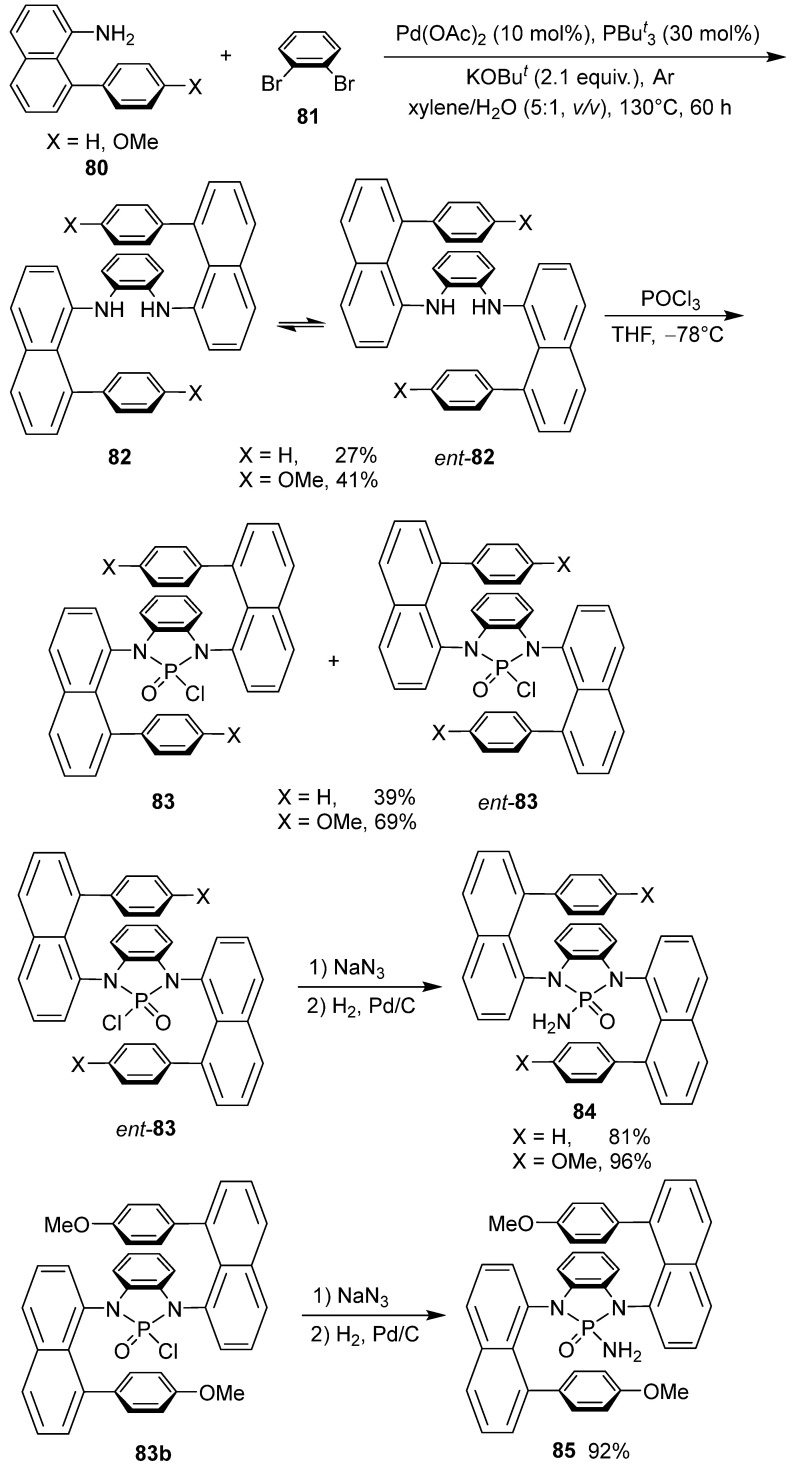
Racemic synthesis of multilayer 3D chiral molecules.

Racemic multilayer 3D chiral molecules were first prepared from 8-arylnaphthalen-1-amines **80** and 1,2-dibromobenzene (**81**) via the palladium-catalyzed dual Buchwald–Hartwig couplings, generating vicinal *N*,*N*’-bis(8-arylnaphthalen-1-yl)benzene-1,2-diamines **82** and *ent*-**82**, which exist in an equilibrium of two enantiomeric conformers **82** and *ent*-**82**. However, after the treatment with phosphorus oxychloride in THF, they cyclized into 2-chloro-1,3-bis(8-arylnaphthalene-1-yl)-1,3-dihydrobenzo[*d*][1,3,2]diazaphosphole 2-oxides **83** and *ent*-**83**, existing in two stable enantiomers 83 and *ent*-83, which were resoluted. They were further converted to 2-amino-1,3-bis(8-arylnaphthalene-1-yl)-1,3-dihydrobenzo[*d*][1,3,2]diazaphosphole 2-oxides **84** and **85**, respectively, under sequential treatments with sodium azide and hydrogenolysis in the presence of Pd/C (Scheme 16). 

**Scheme 17:**
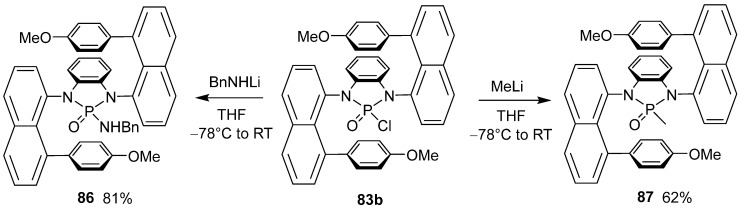
Derivatization of 2-chloro-1,3-bis(8-(4-methoxyphenyl)naphthalene-1-yl)-1,3-dihydrobenzo[*d*][1,3,2]diazaphosphole 2-oxide.

2-Chloro-1,3-bis(8-(4-methoxyphenyl)naphthalene-1-yl)-1,3-dihydrobenzo[*d*][1,3,2]diazaphos-phole 2-oxide (**83b**) was also transformed into 2-benzylamino-1,3-bis(8-(4-methoxyphenyl)naphthalene-1-yl)-1,3-dihydrobenzo[*d*][1,3,2]diazaphosphole 2-oxide (**86**) and 2-methyl-1,3-bis(8-(4-methoxyphenyl)naphthalene-1-yl)-1,3-dihydrobenzo[*d*][1,3,2]diazaphosphole 2-oxide (**87**), respectively, through the reaction with lithium benzylamide and methyl lithium (Scheme 17) [59]. 

**Scheme 18:**
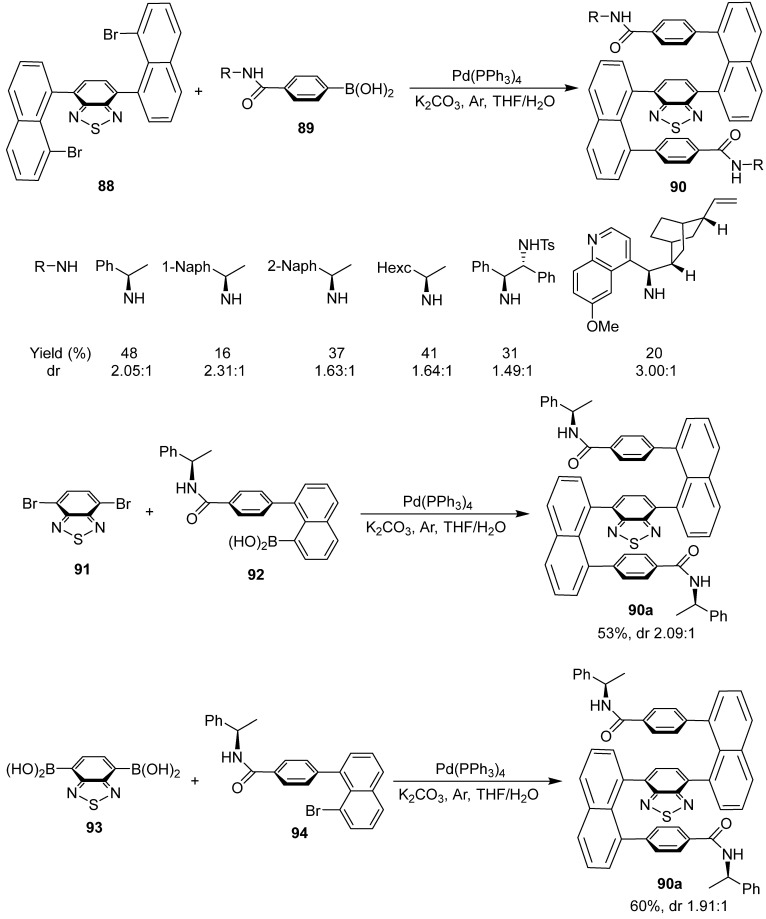
Enantioselective synthesis of multilayer 3D chiral molecules.

In the same year, two different strategies for the enantioselective assembly of multilayer 3D chiral compounds were exploited as well. In the first one, 4,7-bis(8-bromonaphthalen-1-yl)benzo[*c*][1,2,5]thiadiazole (**88**) as a core molecule was reacted with various (*R*)-4-carbamoylphenylboronic acids **89**, respectively, via the palladium-catalyzed dual Suzuki–Miyaura coupling to give rise to the desired products **90** in low 16–48% yields in diastereomeric ratios of 1.49:1 to 3.00:1. To improve the yields, the second strategy was attempted. 4,7-Dibromobenzo[*c*][1,2,5]thiadiazole (**91**) as the central molecule was reacted with (*R*)-(8-(4-((1-phenylethyl)carbamoyl)phenyl)naphthalene-1-yl)boronic acid (**92**) to generate the target product **90a** in 53% yield with a diastereomeric ratio of 2.09:1. Alternatively, the reaction of benzo[*c*][1,2,5]thiadiazole-4,7-diyldiboronic acid (**93**) and (*R*)-4-(8-bromonaphthalen-1-yl)-*N*-(1-phenylethyl)benzamide (**94**) gave rise to the expected product **90a** in 60% yield with a diastereomeric ratio of 1.91:1. The yield was improved slightly, but diastereoselectivity decreased (Scheme 18) [60]. 

**Scheme 19:**
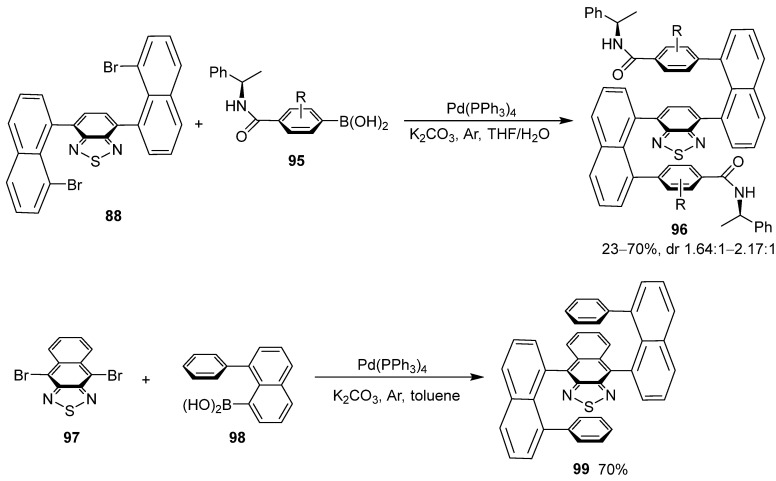
Stereoselective synthesis of multilayer 3D chiral molecules.

To increase the steric bulkiness, different substituted (*R*)-4-(1-phenylethyl)carbamoylphenylboronic acids **95** were applied in the Suzuki–Miyaura coupling with 4,7-bis(8-bromonaphthalen-1-yl)benzo[*c*][1,2,5]thiadiazole (**88**) as a core molecule, giving the desired products **96** in 20–73% yields with diastereomeric ratios of 1.64:1 to 2.17:1. The reaction of 4,9-dibromonaphtho [2,3-*c*][1,2,5]thiadiazole (**97**) and (8-phenylnaphthalen-1-yl)boronic acid (**98**) led to the target product **99** in 70% yield (Scheme 19) [60]. The results indicate that the synthetic strategies involving difunctionalized planar aromatic (hetero)arenes as the central molecules generally result in the formation of the desired products in high yields.

**Scheme 20:**
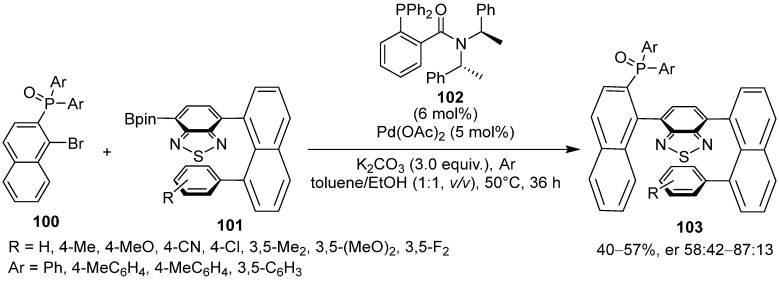
Asymmetric catalytic synthesis of multilayer 3D chiral molecules.

Asymmetric catalytic Suzuki–Miyaura coupling was also developed with diaryl(1-bromonaphthalene-2-yl)phosphine oxides **100** and (7-(8-arylnaphthalen-1-yl)benzo[*c*][1,2,5]thiadiazol-4-yl)boronic acid pinacol esters **101** as starting materials, generating the target products **103** in 40–57% yields with diastereomeric ratios of 58:42 to 87:13 with enantiopure phosphine **102** as chiral ligand (Scheme 20) [61]. Similar asymmetric catalytic Suzuki–Miyaura coupling was also investigated for the synthesis of desired products **96** with a naphthyl group in the middle part instead of the aryl group by displacement of the 8-aryl group with the 8-(naphthalen-1-yl) group in substrates **101** [62]. 

**Scheme 21:**
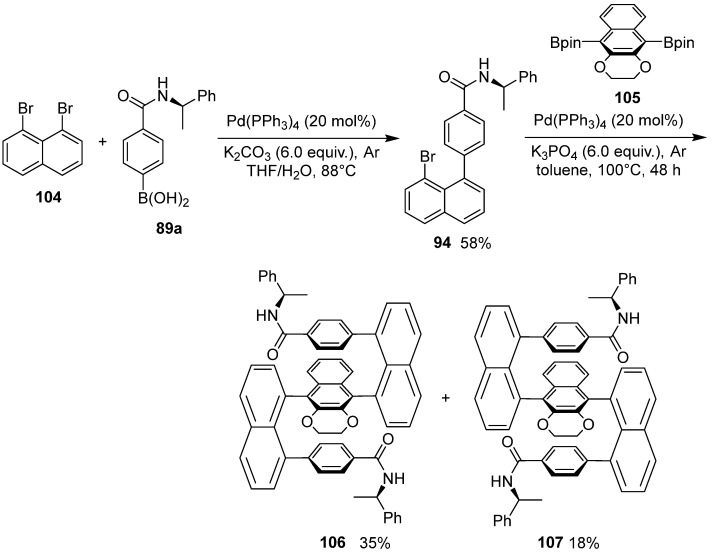
Asymmetric synthesis of multilayer 3D chiral molecules with arenediboronate as the central moiety.

Chiral amide (*R*)-4-(8-bromonaphthalen-1-yl)-*N*-(1-phenylethyl)benzamide **94** was prepared in a 58% yield from 1,8-dibromonaphthalene (**104**) and (*R*)-(4-((1-phenylethyl)carbamoyl)phenyl)boronic acid (**89a**) via the Suzuki–Miyaura coupling. It further dually coupled with pinacol 2,3-dihydronaphtho[2,3-*b*][1,4]dioxine-5,10-diyldiboronate (**97**) to generate a pair of diastereomeric products **106** and **107** in 2:1 in a 53% total yield. The XRD single crystal diffraction analysis reveals that two naphthalenes are located in opposing directions at a dihedral angle of approximately 60°. For the middle part, the top and bottom layers are nearly parallel to the central layer due to the π-stacking interaction. The phenyl group of one of the amide groups is oriented nearly parallel to the naphthalene ring. In a unit cell, the intermolecular distances between proximate aromatic rings are very similar to those of intramolecular distances due to the π-stacking interaction. The functional group amide was further transferred to nitrile, amide, carbamate, amino, and hydroxyl groups, sequentially (Scheme 21) [63].

**Scheme 22:**
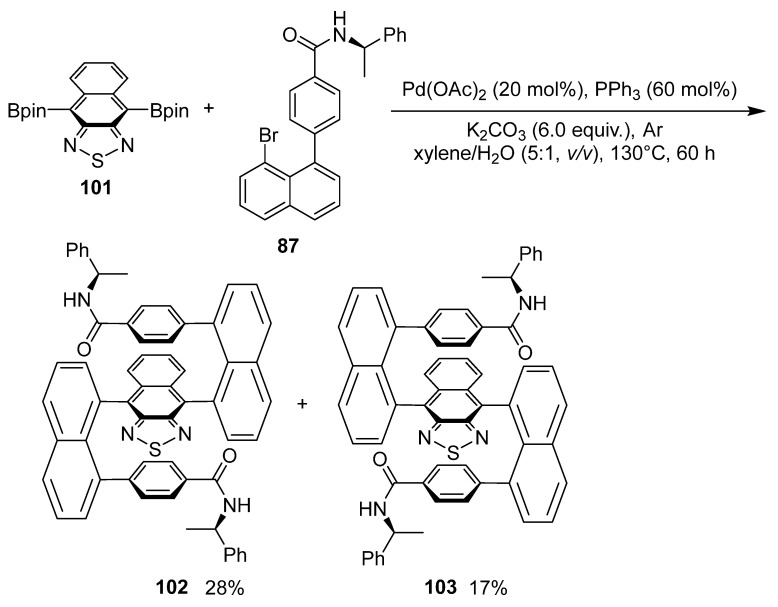
Asymmetric synthesis of multilayer 3D chiral molecules with heteroarenediboronate as the central moiety.

Similarly, chiral amide (*R*)-4-(8-bromonaphthalen-1-yl)-*N*-(1-phenylethyl)benzamide (**94**) was dually coupled with pinacol naphtho[2,3-*c*][1,2,5]thiadiazole-4,9-diyldiboronate (**108**) to generate a pair of diastereomeric products **109** and **110** in 1.6:1 in a 45% total yield. The XRD single crystal diffraction analysis indicates that two naphthalenes are located in opposing directions nearly perpendicular to the central anphthothiadiazole ring, different from the previous 2,3-dihydronaphtho[2,3-*b*][1,4]dioxine central one, which exists at a dihedral angle of approximate 60°. Other structural features are very similar to the previous 2,3-dihydronaphtho[2,3-*b*][1,4]dioxine central one. The functional group amide was further transferred to nitrile, amide, and carbamate groups, sequentially (Scheme 22) [64].

**Scheme 23:**
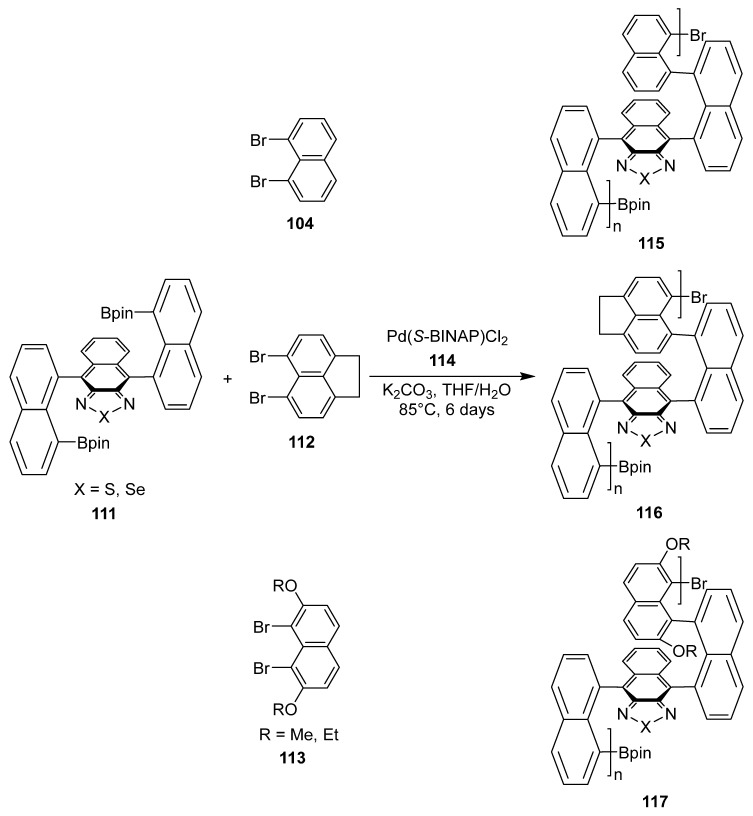
Asymmetric catalytic synthesis of polymeric multilayer 3D chiral molecules.

Recently, a similar synthetic strategy was utilized in the synthesis of polymeric multilayer 3D chiral compounds **115**–**117** with pinacol (naphtho[2,3-*c*][1,2,5]thiadiazole-4,9-diyl)bis(8-naphthalen-1-ylboronate) and (naphtho[2,3-*c*][1,2,5]selenodiazole-4,9-diyl)bis(8-naphthalen-1-ylboronate) **104** with three different 1,8-dibromonaphthalene derivatives **104**, **112**, and **113** as monomers (Scheme 23) [65]. They are very interesting polymers and oligomers of multilayer 3D chiral compounds and show potential application in materials science.

## 7. Conclusions

As one of the most important intramolecular and intermolecular noncovalent interactions in organic chemistry, the π-stacking interaction can exist in different fashions, for instance, in face-to-face, edge-to-face, and even T-shape interactions. It exists widely, such as in biological macromolecules, organic materials, and organic reactions. It plays an important role not only in the stabilization of biological macromolecules and complexations of biomolecules with small organic compounds but also in the stabilization of conformations and transition states in organic reactions via intramolecular and intermolecular attractive interactions. This minireview summarized the recently developed examples of π-stacking interaction-controlled asymmetric synthesis, including auxiliary-induced asymmetric synthesis, kinetic resolution for asymmetric synthesis, diastereoselective synthesis, the asymmetric synthesis of helicenes and heterohelicenes, and the synthesis of multilayer 3D chiral molecules. The π-stacking interaction has been applied in the stabilizations of biomacromolecules, complexations of biomacromolecules and small organic compounds, design of organic materials, organocatalysts, and chiral ligands for asymmetric catalysis. It will show wide applications in understanding the biological function of biomacromolecules and the development of medicines in the future. Before, steric hindrance was considered to be one of the most crucial issues in the design of chiral auxiliaries and catalysts. Recently, steric hindrance, electronic effect, and noncovalent interaction have been recognized as important factors in realizing high stereoselectivity in the design of chiral auxiliaries and catalysts. Recently, several highly efficient asymmetric catalytic reactions have been achieved through the π-stacking interaction between substrates with organocatalysts or chiral ligands under the catalysis of organocatalysts [66,67,68,69,70] and transition metal–chiral ligand complexes [71,72,73], respectively. The enantiomerization of [5]helicene was also successful under the catalysis of a perylene bisimide cyclophane through the π-stacking interaction [74]. As one of the most important intramolecular and intermolecular noncovalent interactions in organic chemistry, the π-stacking interaction will be paid much attention to in the design of novel chiral auxiliaries for stereocontrol in asymmetric synthesis and in the design of new organocatalysts and chiral ligands for asymmetric catalysis in the preparation of biologically important organic compounds, medicines, and their intermediates in the future.

## Data Availability

The data underlying this study are available in the published article.
